# White matter hyperintensities had a correlation with the cerebral perfusion level, but no correlation with the severity of large vessel stenosis in the anterior circulation

**DOI:** 10.1002/brb3.2932

**Published:** 2023-03-14

**Authors:** Fanfan Feng, Weihao Kan, Hongchao Yang, Hongmei Ding, Xiaolong Wang, Ruiguo Dong

**Affiliations:** ^1^ Clinical College, Xuzhou Medical University Xuzhou China; ^2^ Department of Neurology The Affiliated Hospital of Xuzhou Medical University Xuzhou China

**Keywords:** anterior circulation large vessel stenosis, CT angiography, CT perfusion, Fazekas scale, white matter hyperintensity

## Abstract

**Objective:**

The contribution of large vessel stenosis to the development of white matter hyperintensities (WMHs) has not been fully elucidated. This study aims to explore the correlation between ipsilateral white matter hyperintensities (WMHs) and the severity of large vessel stenosis in the anterior circulation and cerebral perfusion level, as well as analyze the factors influencing WMHs.

**Methods:**

A cross‐sectional study of 150 patients with unilateral anterior circulation large vessel stenosis of ≥50% was conducted. The severity of ipsilateral WMHs was assessed by Fazekas scale on T2‐weighted image and/or fluid‐attenuated inversion recovery MR imaging, vascular stenosis severity was evaluated on computed tomography angiography images, and the level of cerebral perfusion was rated according to a staging system for abnormal cerebral perfusion based on CTP results. The relationships between the stenosis severity, cerebral perfusion level and ipsilateral WMHs severity were analyzed. A multivariate logistic regression analysis was performed to determine the factors independently influencing WMHs.

**Results:**

Among 150 patients (mean age, 63.12 ± 10.55 years), there was a statistically significant positive correlation between cerebral perfusion level and the severity of DWMHs and PWMHs (Gamma = 0.561, *p* < .001; Gamma = 0.600, *p* < .001), and a positive correlation between cerebral perfusion level and the severity of vascular stenosis (Gamma = 0.495, *p* < .001).While, there was no statistically significant correlation between the severity of vascular stenosis and the severity of DWMHs and PWMHs (Gamma = 0.188, *p* = .08; Gamma = 0.196, *p* = .06). The multivariate logistic regression analysis results demonstrated that age (OR = 1.047, 95% CI 1.003–1.093; *p* = .035), stroke/TIA history (OR = 2.880, 95% CI 1.154–7.190; *p* = .023) and stage II of cerebral perfusion (OR = 2.880, 95% CI 1.154–7.190; *p* = .023) were independent influencing factors on ipsilateral DWMHs. Age (OR = 1.051, 95% CI 1.009–1.094; *p* = .018), and stage II of cerebral perfusion (OR = 12.871, 95% CI 3.576‐46.322; *p* < .001) were factors independently influencing ipsilateral PWMHs.

**Conclusion:**

White matter hyperintensities may be attributed to cerebral hypoperfusion secondary to vascular stenosis but not directly to the severity of stenosis in the large vessels of anterior circulation. Moreover, longitudinal studies with sequential imaging exams may further reveal the impact of cerebral perfusion secondary to vascular stenosis on the development and progression of WMHs.

## Graphical abstract

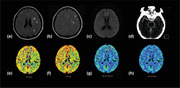

## 1 INTRODUCTION

White matter hyperintensities (WMHs) appear as multiple dotted, patchy or fusion hyperintensities in periventricular or subcortical white matter on T_2_‐weighted imaging (T_2_WI) or fluid‐attenuated inversion recovery (FLAIR), and isointensities or slightly low intensities on T_1_‐weighted imaging (T_1_WI) (Hanning et al., 2019). WMHs are pathological manifestations of demyelination, gliosis, lipid hyaloplasm hyperplasia, and fibrinoid necrosis (Lin et al., 2017), and they occur frequently in patients with vascular risk factors and cerebrovascular diseases (Marek et al., 2018). WMHs are related to cognitive decline, dementia, dyskinesia, abnormal gait, urination dysfunction, increased risk of bleeding after thrombolysis, and recurrence of ischemic stroke (Kim et al., 2014; Rensma et al., 2018; Wang et al., 2017).

Although the pathogenesis of WMHs remains unclear, cerebral ischemia and hypoperfusion may be important mechanisms for the occurrence of WMHs (Wong et al., 2019). From a hemodynamics standpoint, intracranial and extracranial large vessels are located in the “upstream” of small cerebral vessels. The local abnormal blood flow caused by stenosis of large cerebral vessels will be transmitted to the distal end, which may lead to chronic hypoperfusion injury of small cerebral vessels and brain parenchyma, thus resulting in WMHs (Wu et al., 2021). The influence of the severity, distribution, and number of vascular stenosis on WMHs has been evaluated in many studies. The results demonstrate that WMHs are related to stenosis of large arteries, but this conclusion remains controversial (Baradaran et al., 2017; Duan et al., 2018; Nam et al., 2017; Park et al., 2015) .It is considered that hypoperfusion plays an important role in WMHs caused by vascular stenosis (Stewart et al., 2021; Wang et al., 2020; Wong et al., 2019), but there is a lack of research on the mechanism of cerebral perfusion after vascular stenosis affecting WMHs. CT perfusion (CTP) technique can be employed to semi‐quantitatively obtain the blood perfusion volume of local brain tissues and accurately reflect the changes in the blood perfusion volume of brain tissues. Ren et al. (2021) and Yin et al. (2018) proposed a staging system for abnormal cerebral perfusion based on CTP results, which can distinguish different perfusion states and provide guidance for clinicians to select proper therapeutic regimens and evaluate the prognosis for patients with cerebral ischemia. This staging system was adopted in this study to evaluate the cerebral perfusion level. In this study, we aimed to investigate the relationships between the anterior circulation large vessel stenosis severity, cerebral perfusion level and ipsilateral WMHs severity, we hypothesized that the severity of WMHs may be related to cerebral hypoperfusion secondary to vascular stenosis. This may improve our understanding of how vascular stenosis affects the presence or progression of WMHs and may provide an important theoretical basis for the prophylaxis and treatment of WMHs.

## 2 RESEARCH SUBJECTS AND METHODS

### 2.1 Research subjects

Patients who were admitted to the Neurology Department of the Affiliated Hospital of Xuzhou Medical University from July 2018 to January 2022 were retrospectively included in this study. The inclusion criteria were as follows: (1) patients with unilateral anterior circulation large vessel stenosis ≥ 50% on CTA, including stenotic lesions in the internal carotid artery, middle cerebral artery, and anterior cerebral artery; (2) patients with complete data of head MRI, cerebral CTP, and head and neck CTA (The time interval between the three examinations was less than or equal to 1 week) (Ren et al., 2021); (3) patients with complete clinical data. The exclusion criteria included: (1) patients with new strokes, history of severe traumatic brain injury or undergoing cerebrovascular surgery; (2) patients with nonischemic white matter lesions, such as intracranial tumors, multiple sclerosis, optic neuromyelitis, and metabolic diseases; (3) patients with other vascular diseases (such as vasculitides, aneurysms, cerebral arteriovenous malformations, and arterial dissections); (4) patients with serious medical diseases, such as acute coronary syndrome, respiratory failure, severe autoimmune diseases, severe infections, and liver and kidney failure, and patients receiving radiotherapy and chemotherapy. This study was approved by the Ethics Committee of Xuzhou Medical University.

### 2.2 Baseline data collection

The baseline data included age, gender, and medical history of hypertension, diabetes, coronary atherosclerotic heart disease, stroke/(transient ischemia attack)TIA, atrial fibrillation, alcohol abuse, and smoking.

### 2.3 Imaging examinations and relevant diagnostic criteria

#### 2.3.1 Head MRI examination and WMH grading

WMHs were depicted as hyperintensities on T_2_WI and FLAIR, and isointensities or slightly low intensities on T_1_WI. WMHs were divided into periventricular WMHs (PWMHs) and deep WMHs (DWMHs) based on the distance between lesions and the lateral ventricular wall, both of which were scored by the Fazekas scale, respectively. PWMHs were scored as follows: 0 points, no lesions; 1 point, cap‐shaped or pencil‐like thin‐layer lesions; 2 points, smooth halo‐like lesions; 3 points, irregular paraventricular hyperintensities. DWMHs were scored as follows: 0 points, no lesions; 1 point, punctate small lesions; 2 points, initially fused lesions; 3 points, extensively fused lesions. 1 point represented mild WMHs, 2 points represented moderate WMHs, and 3 points represented severe WMH. The total score (0–6 points) was the sum of PWMH and DWMH scores (Ryu et al., 2019).

#### 2.3.2 Evaluation of anterior circulation large vessel stenosis

Head and neck CTA examinations were performed with 256‐slice spiral CT (Philips iCT). The scanning was performed under the following parameters: Tube voltage of 120 kV, effective milliampere‐second of 200 mAs, collimator width of 64 mm × 0.6 mm, the pitch of 1.0, rotation speed of 0.4 s, original image acquisition slice thickness of 1.0 mm, reconstruction slice thickness of 0.5 mm, and slice spacing of 0.5 mm. Based on the calculation method related to warfarin‐aspirin symptomatic intracranial disease (WASID) (Feng et al., 2021), the severity of stenosis for intracranial arteries = [1 – (the diameter of the artery at the most severe stenosis site/the diameter of the normal artery)] × 100%. According to the standard in the North American Symptomatic Carotid Endarterectomy Trial (NASCET) (Feng et al., 2021), the severity of stenosis for extracranial arteries = [(the diameter of the artery above the stenosis site – the diameter of nonstenosis at the most severe stenosis site)/the diameter of the artery above the stenosis site] × 100%. According to the severity of stenosis, the patients were assigned into three groups: moderate, 50% to 69%; severe, 70% to 99%; occlusion, 100%.

#### 2.3.3 Evaluation of cerebral perfusion

The 256‐slice spiral CT (Philips iCT) was used to perform scanning examinations. The 6‐cm layer adjacent to the basal ganglia was selected. The scanning was performed under tube voltage of 80 kV and tube current of 150 mAs. During the examination, 50 mL of iohexol (iodine content: 350 mg/mL) was injected from the median cubital vein with a high‐pressure syringe at a flow rate of 5.0 mL/s, and then flushed with 50 mL of normal saline. Subsequently, 60 s of continuous dynamic scanning was started with a delay of 5 s and a sampling interval of 2 s. As a result, the images with 480 frames were obtained. Standard reconstruction was performed under the slice thickness of 4 mm, the slice spacing of 4 mm, and the FOV of 190 mm × 190 mm. The postprocessing of cerebral perfusion imaging was performed by CT Brain Perfusion on the Philips IntelliSpace Portal workstation (v6.0. 5.02900). The pseudo‐color images related to such perfusion parameters as cerebral blood volume (CBV), cerebral blood flow (CBF), mean transit time (MTT), and time to peak (TTP) were obtained. The region of interest (ROI) was plotted on the perfusion image after avoiding blood vessels and calcification. The lesion area and contralateral mirror area parameters were automatically generated by software to perform a quantitative analysis. Based on the staging system proposed by Yin et al. (2018), abnormal cerebral perfusion was divided into four stages, including Stage I_1_: prolonged TTP and normal MTT, CBF, and CBV; Stage I_2_: prolonged TTP and MTT, normal CBF, and normal or slightly increased CBV; Stage II_1_: prolonged TTP and MTT, decreased CBF, and basically normal or slightly decreased CBV; Stage II_2_: prolonged TTP and MTT, decreased CBF, and decreased CBV. Stage I included stage I_1_ and stage I_2_. Stage II included stage II_1_ and stage II_2_.

All the above images were independently interpreted by two senior neuroimaging doctors. The third doctor would participate in discussion and consultation in case of any disagreement. There was excellent interobserver agreement in evaluating the vascular stenosis, DWMHs, PWMHs, and cerebral perfusion with kappa values of 0.856, 0.874, 0.893, and 0.866, respectively.

### 2.4 Statistical analysis

Statistical analysis was performed by the SPSS 26.0 software. Normally distributed data were expressed as mean ± standard deviation ($\bar{x}\;$± *s*), and an independent sample t‐test was performed to compare these data between groups. The data that did not conform to a normal distribution were expressed by median (quartile). The classification and counting data were expressed by the number of cases (percentage), and the χ^2^ test was performed to compare these data between groups. The relationships between the stenosis severity, cerebral perfusion level and ipsilateral WMHs severity were tested by Gamma correlation analysis, as they are both ordinal level variables. Logistic regression analysis enabled the analysis of factors influencing WMHs. The Wilcoxon signed‐rank test was applied to evaluate the differences in WMH scores between the ipsilateral hemisphere of vascular stenosis and the contralateral hemisphere. *p* < .05 indicated the threshold for a statistically significant difference.

## 3 RESULTS

### 3.1 Demographic and clinical characteristics of patients

**FIGURE 1:**
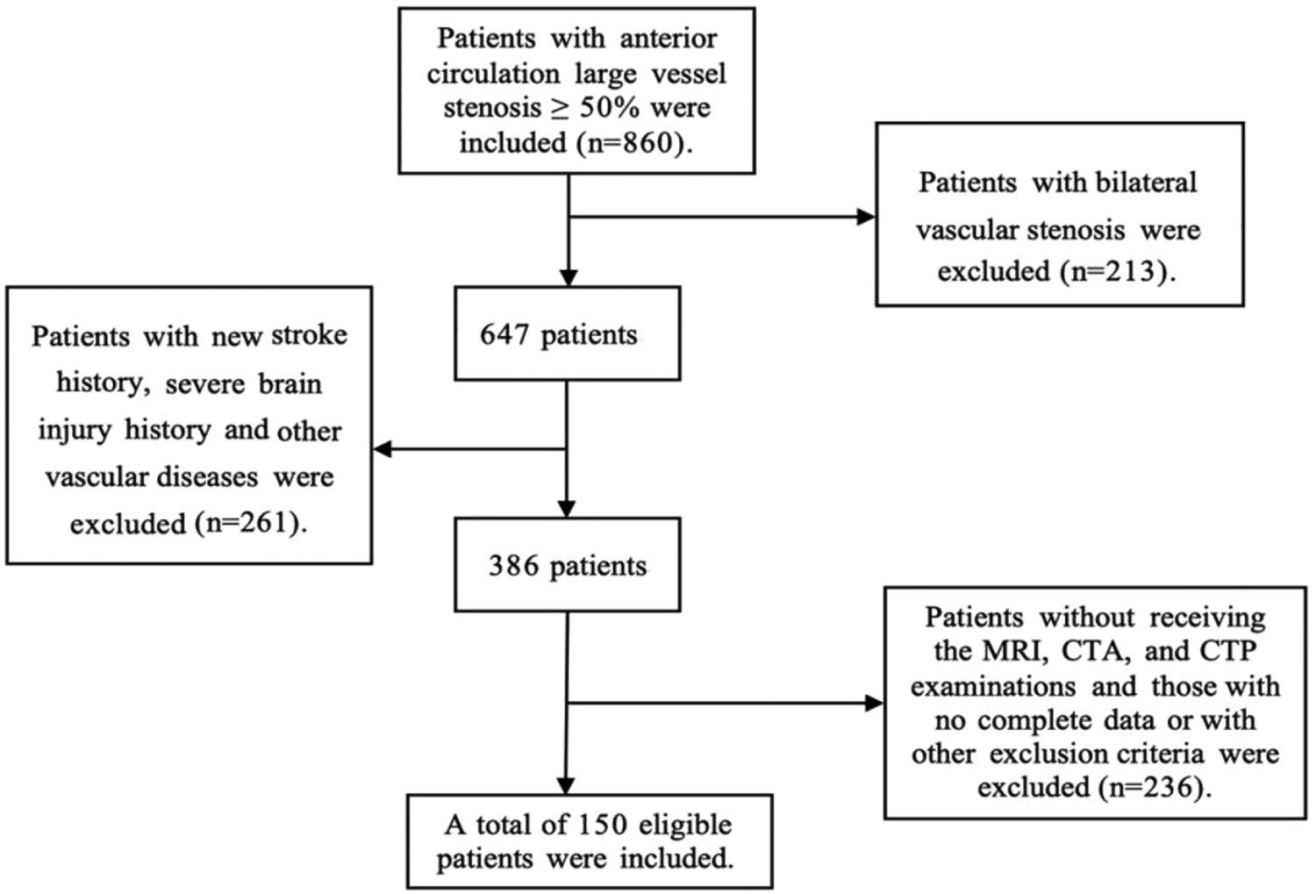
The enrollment flowchart.

According to the inclusion and exclusion criteria (refer to the enrollment flow chart for details; Figure 1), a total of 150 patients (with an average age of 63.12 ± 10.55 years old) were included in this study. Table 1 illustrates the demographic and clinical characteristics of these patients. Among them, 100 patients (66.7%) were male and 50 (33.3%) were female. Besides, 92 patients (61.3%) suffered from hypertension, 52 (34.7%) had stroke/TIA history, and 51 (34.0%) had a history of smoking. Eighty‐six (57.3%) patients had severe vascular stenosis, and 85 patients (56.7%) were in stage II of cerebral perfusion. The age and Hcy values of patients in the moderate‐severe DWMH group were higher than those in the nonmild DWMH group (*p* < .05) (Table 2). Among them, the proportion of patients with a smoking history, hypertension, stroke/TIA history or stage II of cerebral perfusion in the moderate‐severe DWMH group was significantly higher than in the nonmild DWMH group (*p* < .05) (Table 2). Additionally, patients with moderate‐severe PWMH were older and had a higher rate of hypertension, stroke/TIA history, and stage II of cerebral perfusion than patients with nonmild PWMH (*p* < .05) (Table 2).

**TABLE 1:** Demographic and clinical characteristics of patients

	Total (*n* = 150)
Age, mean (SD)	63.12 ± 10.55
Male, *n* (%)	100 (66.7)
Smoking, *n* (%)	51 (34.0)
Alcohol drinking, *n* (%)	37 (24.7)
Hypertension, *n* (%)	92 (61.3)
Diabetes, *n* (%)	45 (30.0)
CHD, *n* (%)	23 (15.3)
Stroke/TIA, *n* (%)	52 (34.7)
Atrial Fibrillation, *n* (%)	5 (3.3)
Hyperlipidemia, *n* (%)	35 (23.3)
TC (mmol/L), mean (SD)	4.17 ± 1.19
TG (mmol/L), mean (SD)	1.28 (0.94, 1.98)
HDL (mmol/L), mean (SD)	1.14 ± 0.30
LDL (mmol/L), mean (SD)	2.38 ± 0.93
Hcy (umol/L), mean (SD)	12.26 (10.54, 14.30)
**Severity of vascular stenosis, *n* (%)**	
moderate	42 (28.0)
severe	86 (57.3)
occlusion	22 (14.7)
**Stenosis site, *n* (%)**	
Internal carotid artery	64 (42.7)
Middle cerebral artery	79 (52.7)
Anterior cerebral artery	7 (4.7)
**Cerebral perfusion level, *n* (%)**	
Normal	23 (15.3)
Stage I	42 (28.0)
Stage II	85 (56.7)
**Clinical manifestations**	
Dizziness	56 (37.3)
Discovery of cerebral vascular stenosis	22 (14.7)
Headache	20 (13.3)
Limb weakness/numbness	34 (22.7)
Loss of consciousness	6 (4.0)
Others	12 (8.0)

CHD, coronary heart disease; TIA, transient ischemia attack; TC, total cholesterol; TG, triglyceride; HDL, high density lipoprotein; LDL, low density lipoprotein; Hcy, homocysteine.

**TABLE 2:** Comparison of patient demographics and clinical characteristics between groups

	DWMH			PWMH		
	Nonmild (*n* = 66)	Moderate‐severe (*n* = 84)	*p*	Nonmild (*n* = 80)	Moderate‐severe (*n* = 70)	*p*
Age, mean (SD)	59.76 ± 9.86	65.76 ± 10.38	<.001	60.36 ± 10.83	66.27 ± 9.34	<.001
Male, *n* (%)	42 (63.6)	58 (69.0)	.485	50 (62.5)	50 (71.4)	.247
Smoking, *n* (%)	15 (22.7)	36 (42.9)	.010	25 (31.3)	26 (37.1)	.447
Alcohol Drinking, *n* (%)	13 (19.7)	24 (28.6)	.211	17 (21.3)	20 (28.6)	.299
Hypertension, *n* (%)	34 (51.5)	58 (69.0)	.029	43 (53.8)	49 (70.0)	.041
Diabetes, *n* (%)	17 (25.8)	28 (33.3)	.315	22 (27.5)	23 (32.9)	.475
CHD, *n* (%)	8 (12.1)	15 (17.9)	.333	15 (18.8)	8 (11.4)	.214
Stroke/TIA, *n* (%)	13 (19.7)	39 (46.4)	<.001	22 (27.5)	30 (42.9)	.049
Atrial Fibrillation, *n* (%)	2 (3.0)	3 (3.6)	.855	3 (3.8)	2 (2.9)	.761
Hyperlipidemia, *n* (%)	16 (24.2)	19 (22.6)	.815	20 (25.0)	15 (21.4)	.606
TC (mmol/L), mean (SD)	4.30 ± 1.32	4.06 ± 1.07	.223	4.33 ± 1.30	3.99 ± 1.03	.085
TG (mmol/L), mean (SD)	1.65 ± 1.34	1.59 ± 1.09	.757	1.70 ± 1.26	1.52 ± 1.14	.363
HDL (mmol/L), mean (SD)	1.17 ± 0.33	1.11 ± 0.28	.232	1.14 ± 0.33	1.13 ± 0.27	.771
LDL (mmol/L), mean (SD)	2.50 ± 1.02	2.27 ± 0.84	.174	2.50 ± 1.03	2.21 ± 0.79	.123
Hcy (umol/L), mean (SD)	11.82 (10.15, 13.37)	13.60 (10.56, 15.11)	.003	12.96 ± 5.08	13.62 ± 4.75	.417
**Severity of vascular stenosis, *n* (%)**			.125			.169
moderate	24 (36.4)	18 (21.4)		27 (33.8)	15 (21.4)	
severe	34 (51.5)	52 (61.9)		44 (55.0)	42 (60.0)	
occlusion	8 (12.1)	14 (16.7)		9 (11.3)	13 (18.6)	
**Stenosis site, *n* (%)**			.707			.235
Internal carotid artery	29 (43.9)	35 (41.7)		38 (47.5)	26 (37.1)	
Middle Cerebral artery	33 (50.0)	46 (54.8)		40 (50.0)	39 (55.7)	
Anterior Cerebral artery	4 (6.1)	3 (3.6)		2 (2.5)	5 (7.1)	
**Cerebral perfusion level, *n* (%)**			<.001			<.001
Normal	18 (27.3)	5 (6.0)		18 (22.5)	5 (7.1)	
Stage I	26 (39.4)	16 (19.0)		32 (40.0)	10 (14.3)	
Stage II	22 (33.3)	63 (75.0)		30 (37.5)	55 (78.6)	

CHD, coronary heart disease; TIA, transient ischemia attack; TC, total cholesterol; TG, triglyceride; HDL, high density lipoprotein; LDL, low density lipoprotein; Hcy, homocysteine.

### 3.2 Correlations of the ipsilateral WMH severity with the severity of anterior circulation large vessel stenosis and cerebral perfusion level

**FIGURE 2:**
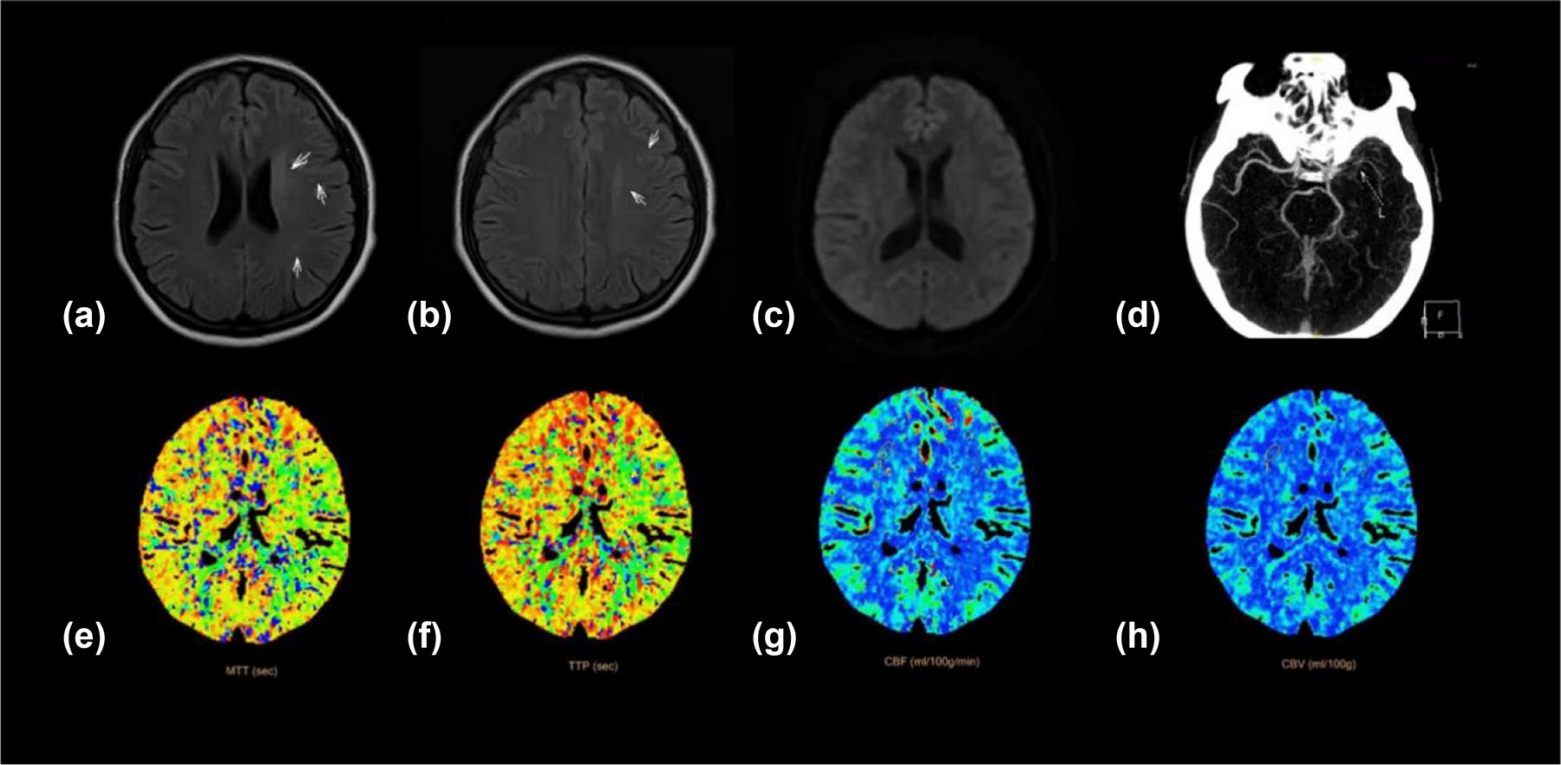
A patient with severe stenosis of the left middle cerebral artery (D), and stage II1 of cerebral perfusion (E, H). The left side has significantly more DWMHs and PWMHs than the right side (A, B); DWI did not show abnormalities (C).

**FIGURE 3:**
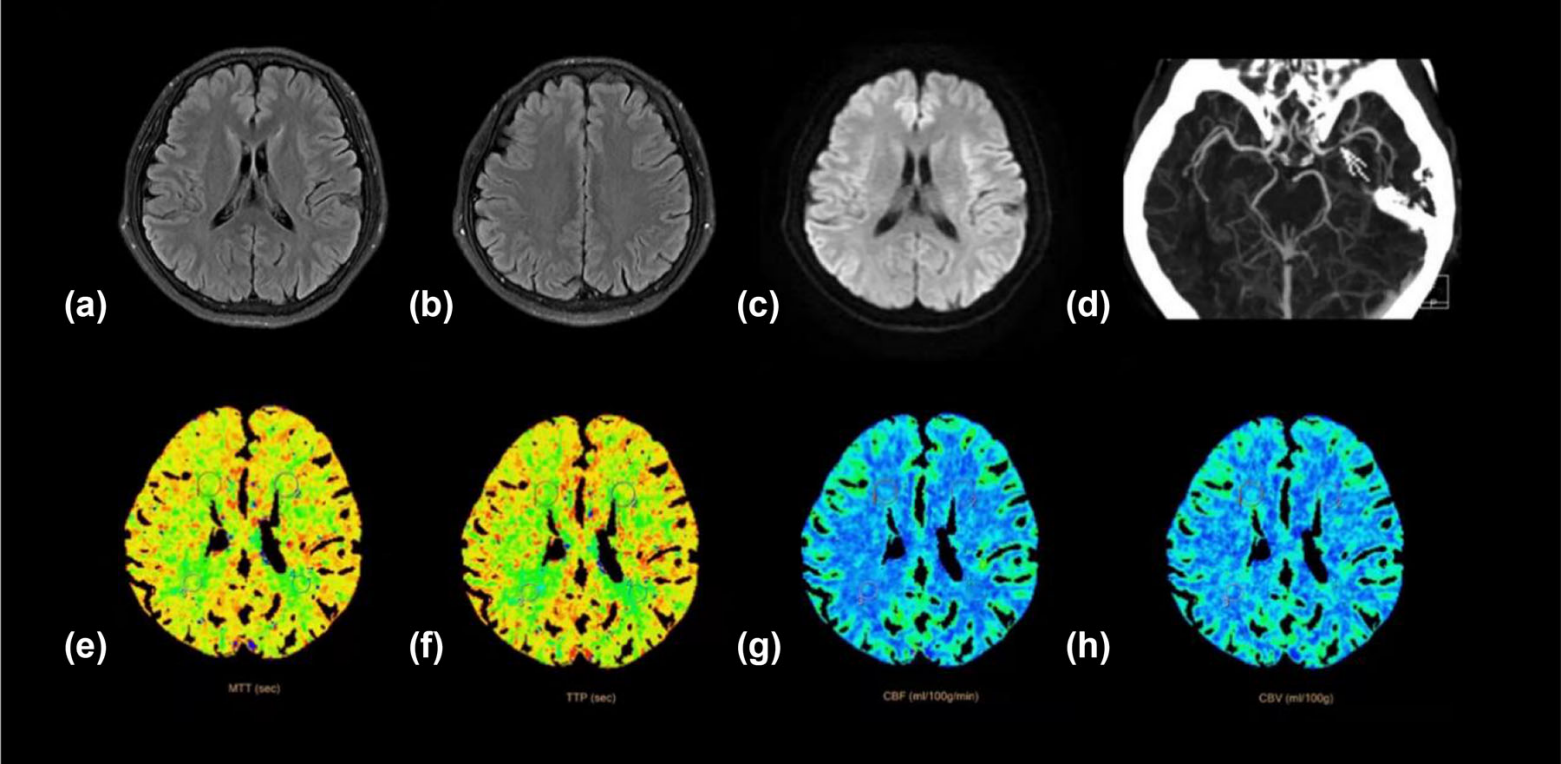
Another patient with severe stenosis of the left middle cerebral artery (D), and normal cerebral perfusion (E, H).There was little difference in WMHs between the two sides (A, B); DWI did not show abnormalities (C).

According to univariable analysis, there was no difference in the severity of stenosis and site of stenosis between patients in the nonmild DWMH and moderate‐severe DWMH groups, as well as in the nonmild PWMH and moderate‐severe PWMH groups (*p* > .05), and the proportion of stage II patients was significantly higher in the moderate‐severe DWMH and moderate‐severe PWMH groups than in the nonmild DWMH and nonmild PWMH groups (*p* < .05) (Table 2). The relationships between the severity of vascular stenosis, cerebral perfusion level, and ipsilateral WMHs severity were tested by Gamma correlation analysis. The results showed that there was a statistically significant positive correlation between cerebral perfusion level and the severity of DWMHs and PWMHs (Gamma = 0.561, *p* < .001; Gamma = 0.600, *p* < .001), and a positive correlation between cerebral perfusion level and the severity of vascular stenosis (Gamma = 0.495, *p* < .001). While, there was no statistically significant correlation between the severity of vascular stenosis and the severity of DWMHs and PWMHs (Gamma = 0.188, *p* = .08; Gamma = 0.196, *p* = .06). Figures 2 and 3 showed images of two cases.

### 3.3 Logistic regression analysis of factors influencing WMHs

In the univariate analysis, age, smoking history, hypertension, stroke/TIA history, Hcy, and cerebral perfusion level were strongly associated with ipsilateral DWMHs. Since *p* < .05 for all of these variables, they were included as covariates in the subsequent binary logistic regression. Age (OR = 1.047, 95% CI 1.003–1.093; *p* = .035), stroke/TIA history (OR = 2.880, 95% CI 1.154–7.190; *p* = .023), and stage II of cerebral perfusion (OR = 2.880, 95% CI 1.154–7.190; *p* = .023) were independent influencing factors on ipsilateral DWMHs. (Table 3). Table 4 shows that older age (OR = 1.051, 95% CI 1.009–1.094; *p* = .018), and stage II of cerebral perfusion (OR = 12.871, 95% CI 3.576‐46.322; *p* < .001) were factors independently influencing ipsilateral PWMHs.

**TABLE 3:** Logistic regression analysis of factors influencing DWMHs

Factor	*B*	SE	Waldχ^2^	*p*	OR (95%CI)
Age	0.046	0.022	4.464	.035	1.047 (1.003–1.093)
Smoking	0.801	0.451	3.153	.076	2.229 (0.920–5.398)
hypertension	0.619	0.446	1.925	.165	1.857 (0.775–4.450)
stroke/TIA history	1.058	0.467	5.139	.023	2.880 (1.154–7.190)
Hcy	0.090	0.049	3.382	.066	1.094 (0.994 –1.205)
Cerebral perfusion level					
Normal				Ref	
Stage I	1.074	0.699	2.356	.125	2.926 (0.743‐11.523)
Stage II	2.555	0.653	15.290	<.001	12.871 (3.576‐46.322)

TIA, transient ischemia attack; Hcy, homocysteine.

**TABLE 4:** Logistic regression analysis of factors influencing PWMHs

Factor	*B*	SE	Waldχ^2^	*p*	OR (95%CI)
Age	0.049	0.021	5.634	.018	1.051 (1.009–1.094)
Hypertension	0.651	0.414	2.477	.116	1.917 (0.852–4.313)
Stroke/TIA history	0.301	0.402	0.558	.455	1.351 (0.614–2.972)
Cerebral perfusion level					
Normal				Ref	
Stage I	0.031	0.655	0.002	.963	1.031 (0.286‐3.722)
Stage II	1.857	0.587	10.008	.002	6.407 (2.027‐20.248)

TIA, transient ischemia attack.

### 3.4 Severity of WMHs on the ipsilateral and contralateral sides of vascular stenosis

The Wilcoxon signed‐rank test on WMH severity between the ipsilateral and contralateral sides of the stenosis revealed that the scores of DWMHs and PWMHs were higher in the ipsilateral hemisphere of stenosis than in the contralateral hemisphere (*p* < .001 for both; Table 5).

**TABLE 5:** Comparison of severity of WMHs on the ipsilateral and contralateral sides of vascular stenosis

	Ipsilateral hemisphere	Contralateral hemisphere	*p*
DWMHs			
Fazekas scale 0	29 (19.3)	65 (43.3)	
Fazekas scale 1	37 (24.7)	47 (31.3)	<.001
Fazekas scale 2	52 (34.7)	26 (17.3)	
Fazekas scale 3	32 (21.3)	12 (8.0)	
PWMHs			<.001
Fazekas scale 0	13 (8.7)	22 (14.7)	
Fazekas scale 1	68 (45.3)	82 (54.7)	
Fazekas scale 2	45 (30)	35 (23.3)	
Fazekas scale 3	24 (16)	11 (7.3)	

## 4 DISCUSSION

In this study, we investigated whether the severity of vascular stenosis and the level of cerebral perfusion were associated with ipsilateral WMH severity. The results revealed that there was a statistically significant positive correlation between cerebral perfusion level and the severity of DWMHs and PWMHs. However, the severity of vascular stenosis was not significantly associated with DWMH and PWMH severity. Logistic regression analysis further confirmed that cerebral hypoperfusion was an independent influencing factor on ipsilateral DWMHs and PWMHs.

In the past, WMHs were generally assumed to be associated with small vascular lesions in the brain. However, nowadays, more academics believe that there is a relationship between WMHs and large vessels in the brain. Wang et al. (2022) performed a retrospective study on 516 patients with intracranial atherosclerosis (ICAS) and found that there was no correlation between WMHs and the severity of large arterial stenosis. Their findings are consistent with the results of this study. Park et al. (2015) demonstrated that the severity of WMH is positively correlated to the severity of intracranial vascular stenosis. Duan et al. (2018) included 2420 patients with acute ischemic stroke or transient ischemic attack in a multicenter study, and they found that WMHs correlated with intracranial arterial stenosis. The inconsistency of these results may be related to differences in cerebral perfusion level after the occurrence of vascular stenosis, since the severity and distribution of vascular stenosis can cause changes in cerebral hemodynamics. However, not all patients with cerebral vascular stenosis would suffer from hypoperfusion due to collateral circulation and other factors (Lan et al., 2020). While most previous studies have assessed overall WMH severity, this study evaluates WMH severity ipsilateral to the stenosis, which helps to explain the pathogenesis of WMHs.

Existing studies on the relationship between WMHs and cerebral macrovascular stenosis focus on stenosis severity, stenosis distribution, and the number of stenoses, while few studies directly refer to cerebral perfusion. Bahrani et al. (2017) and Chen et al. (2018) reported that WMHs increased with decrements in the local cerebral blood flow. Fang et al. (2020) adopted the signal intensity ratio (SIR) to express cerebral hemodynamics. They found that patients with relatively severe middle cerebral artery stenosis were more likely to suffer from worse WMHs in the ipsilateral cerebral hemisphere. Ni et al. (2020) adopted the TTP‐ASPECTS scale to evaluate the cerebral perfusion level based on cerebral CTP examinations. The results indicated that DWMHs were attributed to chronic hypoperfusion of atherosclerotic stenosis. In this study, the cerebral perfusion level of patients with vascular stenosis was explored based on cerebral CTP examinations to investigate the correlation of WMHs with the severity of vascular stenosis and cerebral perfusion level from morphological and functional perspectives.

CTP is a functional imaging technique. The time‐density curve of contrast media passing through tissues for the first time can be obtained by dynamic scanning, and the cerebral perfusion parameters (CBV, CBF, TTP, and MTT) can be determined using the software. CBV is defined as the volume occupied by intravascular blood within a particular quantity of brain tissue (including capillaries and large vessels). A drop in CBV denotes poor cerebral microcirculation. On the other hand, CBF is defined as the amount of blood flow through certain cerebral vascular structures (arteries, capillaries, veins, and venous sinuses) in a unit interval. A decline in CBF delineates an impairment of the cerebral microcirculation. Meanwhile MTT is defined as the average time for contrast media to pass through the ROI. The prolonged MTT indicates decreased cerebral perfusion pressure and impaired perfusion reserve. Similarly, TTP is defined as the time duration from the beginning of injection of contrast media to reaching the peak concentration in the ROI. The prolonged TTP is mainly caused by the slow blood flow or collateral blood supply (Tao et al., 2022; Yang et al., 2022). Comprehensive analysis of these parameters contributes to an in‐depth insight into the blood supply in brain tissues. Based on the staging system proposed by Yin et al. (2018), abnormal cerebral perfusion can be divided into two stages. In Stage I_1_, there is a change in the cerebral blood flow velocity, but no compensatory dilatation of local cerebral microvessels. In Stage I_2_, there is compensatory dilatation in local cerebral microvessels. In Stage II_1_, there is a decrease in CBF and local astrocyte swelling due to ischemia, which begins to compress local microvessels. In Stage II_2_, there is significant astrocyte swelling, which compresses, narrows or blocks local microvessels, thus inducing impairment of the local microcirculation. Cerebral hypoperfusion may cause changes in downstream structures by inducing cell energy imbalance, oxidative stress, inflammatory reaction, endoplasmic reticulum stress, mitochondrial dysfunction and other mechanisms. This would result in endothelial dysfunction, damage of the blood‐brain barrier (BBB), glial activation, and apoptosis, thus leading to the progression of WMHs (Huang et al., 2018; Rajeev et al., 2022).

In this study, we discovered that DWMHs and PWMHs were more severe on the ipsilateral side of the stenosis than on the contralateral side. This may be related to their anatomy since DWMHs are supplied by deep penetrating arteries branching from the intracranial soft meningeal arteries. These arteries have relatively short vascular pathways and it is difficult for ischemia to occur in this region when the collateral circulation is good or perfusion is normal. Thus, ischemic changes only happen when perfusion is inadequate or when cerebral blood flow self‐regulation is reduced. Blood supply to the PWMHs is derived from the choroidal artery of the inferior ventricular artery or from the terminal branches of the striatum arteriosus, which has a sparse or absent anastomosis with vessels originating from the cerebral surface (Song et al., 2021). This study revealed that besides cerebral hypoperfusion, WMHs also correlated with age. These are consistent with the findings in some previous studies (Vedala et al., 2019; Wang et al., 2021). The changes in white matter blood supply caused by arteriosclerosis during aging may be one of the factors contributing to the strong correlation between age and WMHs, and most white matter nerve fibers are myelinated and their lengths shorten dramatically during normal aging, so aging can cause demyelination changes (Yu et al., 2018). Stroke/TIA history was significantly associated with ipsilateral DWMHs, probably because they share the same risk factors and are both associated with cerebral underperfusion (Holmegaard et al., 2018).

The current findings on the impact of cerebral hypoperfusion on the severity of WMHs may have clinical implications for the management of patients with vascular stenosis. Improving cerebral perfusion could be highly beneficial to patients with WMHs and cerebrovascular stenosis. It has been demonstrated in a prospective study (Chuang et al., 2011) that the cerebral perfusion and WMH severity of patients can be optimized after internal carotid artery stent implantation. In clinical work, patients with vascular stenosis and ipsilateral WMH should be given high priority, and we can perform further CTP examinations so as to provide a reliable basis for the treatment of WMHs. However, these conclusions necessitate further verification and validation in future prospective studies.

Limitations of our study: First, this study is a single‐center retrospective study, which could not evaluate the causality of the association between cerebral perfusion and WMHs. Therefore, it is still necessary to conduct multicenter, large‐sample and prospective studies to further verify the findings reported in this study. However, the results of the relationships between the severity of large vessel stenosis, the level of cerebral perfusion, and the severity of ipsilateral WMHs may provide important clues for the direction of future research in this area. Second, most patients in our study were symptomatic, a heterogeneous group with different presentations, so sample selection bias was inevitable, a multicenter study including both symptomatic and asymptomatic patients would reduce this error. Third, many patients without stenosis may also have WMHs, and since few patients without stenosis are clinically assessed for cerebral perfusion, patients without stenosis were excluded from this study. Our study has some strengths. First, we combine CTA with CTP, and the vascular stenosis can be more fully represented morphologically and functionally. Second, two experienced radiologists assessed the severity of stenosis, WMH, and cerebral perfusion without knowledge of clinical demographics, which minimized the expected bias. Third, this is a retrospective study, and it is much easier and cheaper to conduct a review of medical records, and valuable information can often be found this way.

In summary, white matter hyperintensities may be attributed to cerebral hypoperfusion secondary to vascular stenosis but not directly to the severity of stenosis in the large vessels of anterior circulation. We need to actively treat and intervene in factors that cause cerebral hypoperfusion in order to delay the development of WMHs. However, further investigations are required to verify these findings and identify potential pathophysiological mechanisms.

## AUTHOR CONTRIBUTIONS

FF, RD, WK, and HY conceived and designed the study. FF, WK, and HY acquired the data. FF, RD, WK, and HY analyzed and interpreted the data. All authors contributed to the writing of the manuscript, read and approved the final version.

## CONFLICT OF INTEREST STATEMENT

The authors have no conflicts of interest to declare.

### ETHICS STATEMENT

This study was approved by the Ethics Committee of the affiliated Hospital of Xuzhou Medical University.

### PEER REVIEW

The peer review history for this article is available at https://publons.com/publon/10.1002/brb3.2932.

## Data Availability

The date that support the findings of this study are available from the corresponding author upon reasonable request.
